# Vitamin E Increases Antimicrobial Sensitivity by Inhibiting Bacterial Lipocalin Antibiotic Binding

**DOI:** 10.1128/mSphere.00564-18

**Published:** 2018-12-12

**Authors:** Marwa M. Naguib, Miguel A. Valvano

**Affiliations:** a Wellcome-Wolfson Institute for Experimental Medicine, Queen’s University Belfast, Belfast, United Kingdom; b Department of Microbiology and Immunology, Faculty of Pharmacy, Damanhour University, Damanhour, Egypt; c Department of Microbiology and Immunology, University of Western Ontario, London, Ontario, Canada; University of Rochester

**Keywords:** Gram-negative bacteria, antibiotic resistance, chronic infection, cystic fibrosis, intrinsic resistance, lipocalin, vitamin E

## Abstract

Burkholderia cenocepacia is an opportunistic Gram-negative bacterium that causes serious respiratory infections in patients with cystic fibrosis. Recently, we discovered that B. cenocepacia produces the extracellular bacterial lipocalin protein BcnA upon exposure to sublethal concentrations of bactericidal antibiotics. BcnA captures a range of antibiotics outside bacterial cells, providing a global extracellular mechanism of antimicrobial resistance. In this study, we investigated water-soluble and liposoluble forms of vitamin E as inhibitors of antibiotic binding by BcnA. Our results demonstrate that *in vitro*, both vitamin E forms bind strongly to BcnA and contribute to reduce the MICs of norfloxacin (a fluoroquinolone) and ceftazidime (a β-lactam), both of them used as model molecules representing two different chemical classes of antibiotics. Expression of BcnA was required for the adjuvant effect of vitamin E. These results were replicated *in vivo* using the Galleria mellonella larva infection model whereby vitamin E treatment, in combination with norfloxacin, significantly increased larva survival upon infection in a BcnA-dependent manner. Together, our data suggest that vitamin E can be used to increase killing by bactericidal antibiotics through interference with lipocalin binding.

**IMPORTANCE** Bacteria exposed to stress mediated by sublethal antibiotic concentrations respond by adaptive mechanisms leading to an overall increase of antibiotic resistance. One of these mechanisms involves the release of bacterial proteins called lipocalins, which have the ability to sequester antibiotics in the extracellular space before they reach bacterial cells. We speculated that interfering with lipocalin-mediated antibiotic binding could enhance the efficacy of antibiotics to kill bacteria. In this work, we report that when combined with bactericidal antibiotics, vitamin E contributes to enhance bacterial killing both *in vitro* and *in vivo.* This adjuvant effect of vitamin E requires the presence of BcnA, a bacterial lipocalin produced by the cystic fibrosis pathogen Burkholderia cenocepacia. Since most bacteria produce lipocalins like BcnA, we propose that our findings could be translated into making novel antibiotic adjuvants to potentiate bacterial killing by existing antibiotics.

## Abstract (precis)

Bacteria exposed to stress mediated by sublethal antibiotic concentrations respond by adaptive mechanisms leading to an overall increase of antibiotic resistance. One of these mechanisms involves the release of bacterial proteins called lipocalins, which have the ability to sequester antibiotics in the extracellular space before they reach bacterial cells. We speculated that interfering with lipocalin-mediated antibiotic binding could enhance the efficacy of antibiotics to kill bacteria. In this work, we report that when combined with bactericidal antibiotics, vitamin E contributes to enhance bacterial killing both *in vitro* and *in vivo.* This adjuvant effect of vitamin E requires the presence of BcnA, a bacterial lipocalin produced by the cystic fibrosis pathogen Burkholderia cenocepacia. Since most bacteria produce lipocalins like BcnA, we propose that our findings could be translated into making novel antibiotic adjuvants to potentiate bacterial killing by existing antibiotics.

## INTRODUCTION

Antimicrobial resistance is an increasingly serious global health problem underpinned by the emergence of opportunistic, multidrug-resistant bacteria (1–3). It is imperative to find new ways of treating bacterial infections, especially those caused by Gram-negative pathogens (4). We recently discovered that the opportunistic bacterium Burkholderia cenocepacia can resist antibiotics by mechanisms operating extracellularly and induced in response to near-lethal antibiotic concentrations (5, 6). This means that under chemotoxic stress, microbes can fight antibiotics even before they reach bacterial cells. Key molecules involved in this mechanism are the polyamine putrescine and bacterial lipocalins, a highly conserved group of β-barrel-shaped proteins of unknown function (YceI family) produced by more than 5,500 bacterial species (5, 7).

Gram-negative bacterial species belonging to the Burkholderia cepacia complex (8), especially B. cenocepacia, are opportunistic pathogens that cause deleterious chronic respiratory infections in patients with cystic fibrosis (9, 10). Once established, infection by B. cenocepacia is very difficult to eradicate since these bacteria display high levels of intrinsic antibiotic resistance to many different antibiotics (11, 12). We showed previously that B. cenocepacia secretes the extracellular bacterial lipocalin protein BcnA (BCAL3311) upon challenge with different classes of bactericidal antibiotics (5, 7). Lipocalins are a functionally diverse family of small ligand-binding proteins that are common to many organisms, from bacteria to humans (13, 14), and which share a conserved β-barrel architecture (15). Both BcnA and other bacterial lipocalins from different pathogens such as Pseudomonas aeruginosa, Mycobacterium tuberculosis, and Staphylococcus aureus, heterologously expressed in B. cenocepacia, contribute to augment multidrug antibiotic resistance *in vitro* and *in vivo* (7). Further, antimicrobial peptides (e.g., polymyxin B and colistin) and other bactericidal antibiotics (e.g., rifampin, norfloxacin, and ceftazidime) can displace the hydrophobic probe Nile Red bound to purified BcnA, suggesting that this lipocalin binds to a range of bactericidal antibiotics (7).

Liposoluble vitamins E (α-tocopherol) and K2 (menaquinone) can overcome antibiotic resistance mediated by bacterial lipocalins (7), but the mechanism of inhibition was not fully elucidated. We hypothesized that vitamin E reduces antimicrobial resistance by binding to BcnA with higher affinity than antibiotics, thus suppressing the contribution of BcnA to antibiotic resistance. In this study, we investigated the mechanism by which vitamin E sensitizes B. cenocepacia to several different antibiotics. We show that both liposoluble and water-soluble forms of vitamin E, in combination with antibiotics, significantly reduce the MIC levels of several bactericidal antibiotics against B. cenocepacia. Further, the potentiating ability of vitamin E on antibiotics requires BcnA since it is not manifested in mutants unable to produce the lipocalin. Using the wax moth Galleria mellonella infection model, we also demonstrate that treatment with vitamin E and antibiotics after infection resulted in increased survival of infected *Galleria* larvae, suggesting that vitamin E has an adjuvant effect that enhances the effectiveness of various bactericidal antibiotics.

## RESULTS

### *bcnA* is part of a three-gene operon.

**FIG 1:**
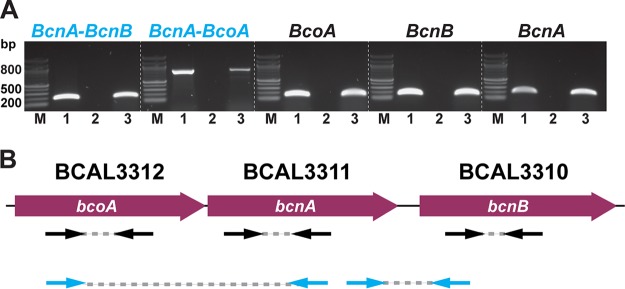
*bcoA*, *bcnA*, and *bcnB* form an operon. (A) PCR amplification using cDNA and gDNA templates derived from B. cenocepacia K56-2. Lanes: M, 100-bp ladder; 1, gDNA-derived PCR product; 2, negative control (no RT during cDNA synthesis); 3, cDNA-derived PCR product. All samples were run in the same gel; the dotted white lines are for clarity only. (B) Intragenic amplicons are in black; intergenic regions are in red. The direction of transcription is indicated. Primers and PCR amplicon positions from panel A are indicated by the black (intragenic regions) and blue (intergenic regions) arrow pairs.

B. cenocepacia K56-2 produces two lipocalin homologues, BcnA (BCAL3311) and BcnB (BCAL3310), of which only BcnA is secreted to the extracellular space and is primarily involved in conferring increased antimicrobial resistance (5, 7). Both *bcnA* and *bcnB* genes in B. cenocepacia are linked to an upstream gene encoding a predicted membrane cytochrome *b*_561_ protein (BCAL3312), which we have annotated as *bcoA* (*bcnA* cytochrome oxidase-associated gene). *bcnA* and *bcoA* are commonly linked loci in many bacterial genomes (e.g., Pseudomonas aeruginosa and *Salmonella*). The genomic organization of the *bcn* region in B. cenocepacia suggests that *bcoA*, *bcnA*, and *bcnB* are cotranscribed (7). To determine if these is the case, we performed RT-PCR assays using primers spanning gene sequences and intergenic regions of these three genes (Fig. 1; see also Table S1 in the supplemental material). PCR amplification of the cDNA templates gave amplicons of the expected sizes comparable to genomic DNA (Fig. 1), while PCR using the negative control failed to give any detectable amplification, indicating that the cDNA samples were clear of genomic DNA contamination (Fig. 1A, lanes 2). These results indicate that *bcoA*, *bcnA*, and *bcnB* form a three-gene operon in B. cenocepacia (Fig. 1B).

10.1128/mSphere.00564-18.3 TABLE S1 Primers used in this study. Download Table S1, PDF file, 0.02 MB. Copyright © 2018 Naguib and Valvano. 2018 Naguib and Valvano This content is distributed under the terms of the Creative Commons Attribution 4.0 International license.

### The effect of vitamin E on B. cenocepacia antibiotic resistance depends on the BcnA protein.

We previously reported that BcnA increases multidrug antibiotic resistance in B. cenocepacia by sequestering bactericidal antibiotics in the extracellular milieu, a property that could be inhibited by liposoluble vitamins like vitamins E and K2 (7). To better characterize the effect of vitamin E on selected antibiotic molecules in the presence or absence of BcnA expression, we performed checkerboard assays using D-α-tocopheryl polyethylene glycol 1000 succinate (TPGS) and several bactericidal antibiotics. In contrast to authentic vitamin E (α-tocopherol), TPGS is a water-soluble vitamin E derivative that arises from the esterification of D-α-tocopheryl succinate with polyethylene glycol (16). We used norfloxacin (a fluoroquinolone), ceftazidime (a β-lactam), and polymyxin B (an antimicrobial peptide) as model bactericidal antibiotics whose activity is affected by BcnA (7). Checkerboard assays demonstrated that 0.7 mM was the most effective concentration of TPGS to work in combination with the antibiotics.

**FIG 2:**
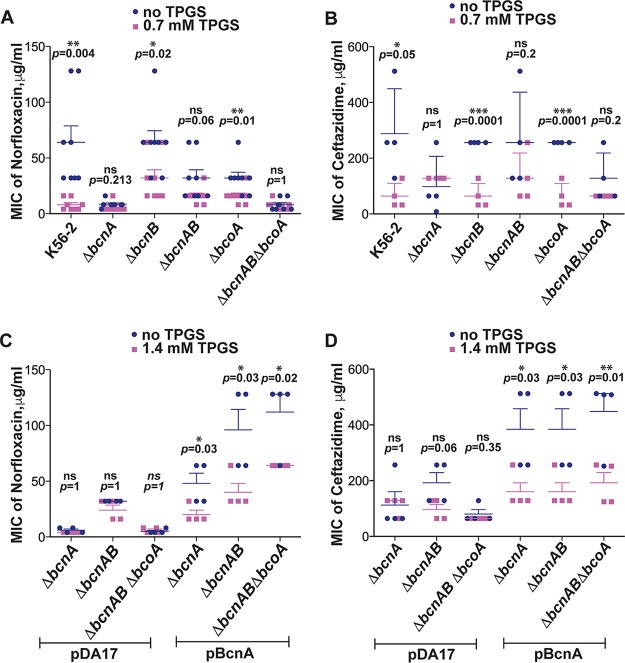
Vitamin E antibiotic adjuvant effect depends on the BcnA protein. (A) MIC of norfloxacin alone or in combination with 0.7 mM TPGS against *Burkholderia* strains, determined by broth microdilution in cation-adjusted Mueller-Hinton broth (MHB) at 24 h. Data points are from 4 independent experiments, each done in duplicate (*n* = 8). (B) MIC of ceftazidime alone or combined with TPGS against *Burkholderia* strains in MHB at 24 h (*n* = 4, from 2 independent experiments). (C) MIC of norfloxacin alone or mixed with 1.4 mM TPGS against Δ*bcnA* mutants carrying pDA17 and Δ*bcnA* mutants harboring pDA17 *bcnA*, determined by broth dilution methods in MHB at 24 h (*n* = 4, from 2 independent experiments). (D) MIC of ceftazidime alone or mixed with 1.4 mM TPGS against Δ*bcnA* mutants carrying pDA17 and Δ*bcnA* mutants harboring pDA17 *bcnA* at 24 h (*n* = 4, from 2 independent experiments). Results are shown as the mean MIC ± SEM, and *P* values were calculated by the paired *t* test.

When TPGS was given together with norfloxacin, there was an 8-fold reduction in the MIC of the parental strain to the antibiotic (MIC of 8 ± 2 μg/ml), compared to bacteria exposed to only norfloxacin (MIC of 64 ± 15 μg/ml; *P = *0.004), while TPGS had no effect on the norfloxacin MIC values (8.5 ± 1.2 μg/ml) of the Δ*bcnA* mutant (Fig. 2A). The lower norfloxacin MIC values for the Δ*bcnA* mutant reflect the reduction in antibiotic resistance associated with the loss of BcnA and in the case of norfloxacin are 8-fold lower than in the parental (*bcnA*^+^) strain (7) and comparable to the MIC of the parental strain in the presence of TPGS. These results indicate that TPGS can reduce the norfloxacin MIC values to similar levels as found in the absence of the *bcnA* gene, suggesting that TPGS directly inhibits BcnA functionality. We also evaluated the role of the other proteins encoded by the *bcoA-bcnAB* operon. The MIC of norfloxacin against the Δ*bcnB* mutant (64 ± 10.5 μg/ml) was reduced to 32 ± 7.4 μg/ml when combined with TPGS (*P = *0.02). Similar results were obtained with norfloxacin-TPGS against the Δ*bcoA* mutant, where the MIC decreased from 32 ± 5.2 μg/ml to 16 ± 3 μg/ml (*P = *0.01) (Fig. 2A). It should be noted that all these mutants were constructed as unmarked nonpolar deletions (7) and therefore contain a functional *bcnA* gene, which explains the lesser effect on MIC values. However, the minor reduction in MIC shown by the Δ*bcoA* mutant may be attributable to the absence of the predicted cytochrome *b*_561_ protein encoded by this gene. The Δ*bcnAB* and Δ*bcnAB* Δ*bcoA* combination mutants had increased sensitivity to norfloxacin alone (MICs of 32 ± 7.4 and 8 ± 2 μg/ml, respectively) (Fig. 2A), and TPGS had no additional effect in reducing these values even further (Fig. 2A), supporting the notion that the TPGS effect is clearly noticeable in the presence of BcnA. Similar results were obtained with ceftazidime (Fig. 2B). In this case, all isolates producing BcnA (strain K56-2 and the Δ*bcnB* and Δ*bcoA* mutants) had MIC values of 288, 256, and 256 μg/ml, respectively, which were 4-fold reduced in combination with TPGS. As with norfloxacin, addition of TPGS had no effect on the MIC against mutants with *bcnA* gene deletions. Therefore, TPGS can contribute to the reduction of MIC levels of two different antibiotics in a BcnA-dependent manner.

We performed complementation experiments to confirm if the observed results were related to the presence of a functional *bcnA* gene. The genetically complemented Δ*bcnA*, Δ*bcnAB*, and Δ*bcnAB* Δ*bcoA* strains, all carrying a plasmid expressing BcnA, exhibited increased antibiotic resistance (mean MICs of norfloxacin of 48, 96, and 112 μg/ml and mean MICs of ceftazidime of 384, 384, and 448 μg/ml, respectively) (Fig. 2C and D). However, the complemented strains had reduced MIC values for norfloxacin and ceftazidime (by approximately >2-fold) when they were treated with 1.4 mM TPGS and antibiotics (Fig. 2C and D), demonstrating that TPGS works through BcnA. We interpreted the higher concentration of TPGS required in these experiments as a consequence of the overexpression of BcnA from a plasmid instead of from the native promoter at the chromosomal location of the *bcnA* gene.

**FIG 3:**
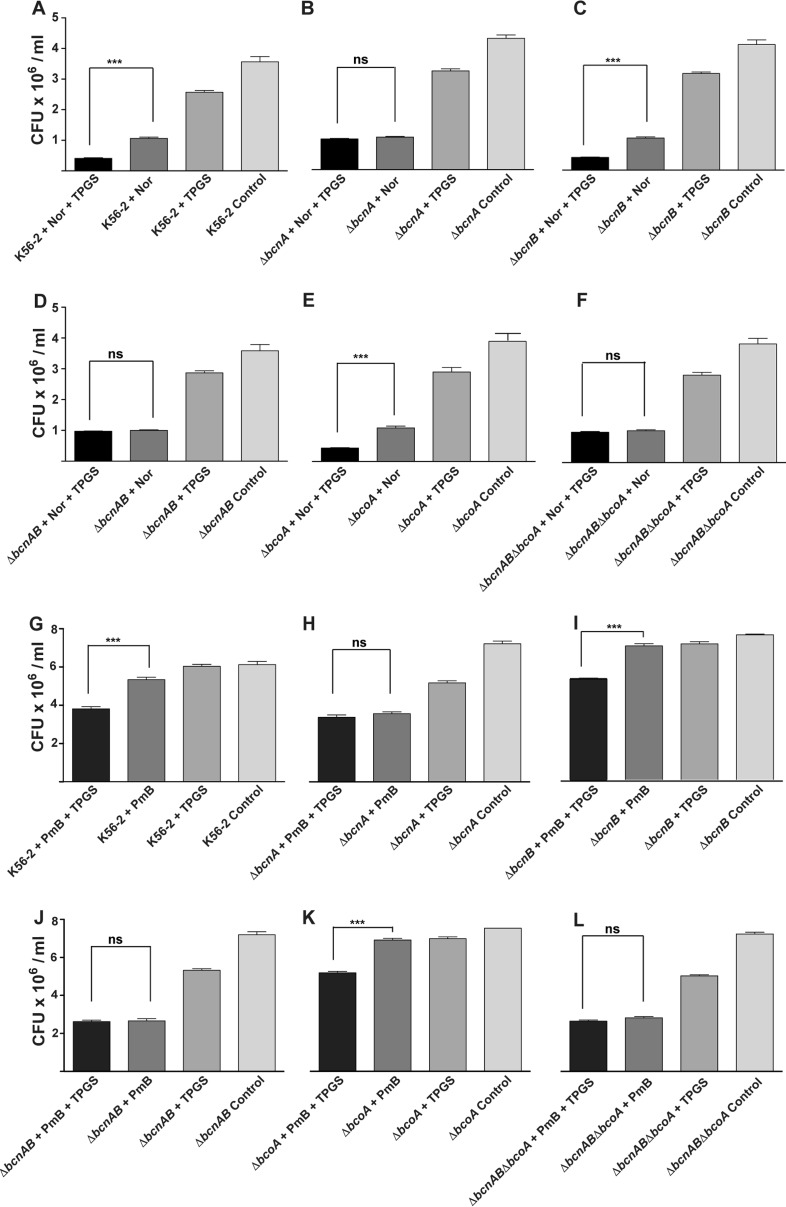
Antibiotic challenge assays. *Burkholderia* strains were challenged with MIC_25_ of norfloxacin, which varied for each strain (16 μg/ml for the parental strain K56-2 and the Δ*bcnB* strain, 8 μg/ml for Δ*bcnAB* and Δ*bcoA* strains, and 2 μg/ml for Δ*bcnA* and Δ*bcnAB* Δ*bcoA* strains) with or without 0.7 mM TPGS (A to F) in cation-adjusted MHB for 2 h at 37°C. For challenge with polymyxin B, bacteria were exposed to MIC_25_ (256 μg/ml of polymyxin B for all strains), with or without 0.7 mM TPGS (G to L), using non-cation-adjusted MHB for 6 h at 37°C. (A and G) Wild-type K56-2; (B and H) Δ*bcnA* strain; (C and I) Δ*bcnB* strain; (D and J) Δ*bcnAB* double mutant; (E and K) Δ*bcoA* strain; (F and L) Δ*bcnAB* Δ*bcoA* triple mutant. Results are shown as the mean MIC ± SEM, by determining the CFU of surviving bacteria. *P* values were calculated by the paired *t* test. Data represent the results of 3 independent experiments, each done in duplicate (*n* = 6).

The observed results did not depend on the vitamin E formulation since liposoluble vitamin E (at 0.2 mM) in combination with norfloxacin also resulted in increased antibiotic activity when the treated strains expressed BcnA or in mutants genetically complemented by introducing the BcnA-encoding plasmid (Fig. S1). Because BcnA becomes important when B. cenocepacia is exposed to subinhibitory concentrations of bactericidal antibiotics (5, 7), we also evaluated the effect of TPGS in bacteria exposed to sub-MIC antibiotic concentrations using in this case norfloxacin and polymyxin B. Subinhibitory antibiotic concentrations were defined as 25% of the MIC (MIC_25_) for each of the strains used (ranging from 2 to 16 μg/ml), and surviving bacteria were enumerated after challenge with norfloxacin and polymyxin B for 2 and 6 h, respectively. Norfloxacin and TPGS in combination caused around a 50% reduction in the number of surviving K56-2 bacteria, compared to norfloxacin alone (Fig. 3A). Similar results were obtained by challenging the Δ*bcnB* and Δ*bcoA* mutant strains (Fig. 3C and E). On the other hand, Δ*bcnA*, Δ*bcnAB*, and Δ*bcnAB* Δ*bcoA* strains showed nearly the same number of bacterial counts as in the norfloxacin challenge alone either in the absence or in the presence of TPGS (Fig. 3B, D, and F). The effect of the antibiotic alone or in combination with TPGS also resulted in significant growth rate reduction over time for the parental K56-2 strain treated with norfloxacin (at MIC_25_) and TPGS compared to treatment with norfloxacin only (Fig. S2A). In contrast, the Δ*bcnA* mutant treated with norfloxacin and TPGS showed no difference in growth rate from treatment with antibiotic alone (Fig. S2B). Challenge experiments with polymyxin B at 256 μg/ml (MIC_25_) and TPGS for 2 h did not show any differences among the strains, likely due to their high level of intrinsic resistance to polymyxin B. However, challenge up to 6 h revealed a significant reduction in the number of surviving bacteria relative to challenge with polymyxin B alone for the parental K56-2 and the Δ*bcnB* and Δ*bcoA* mutant strains (Fig. 3G, I, and K) but no significant changes in the absence of BcnA protein, irrespective of the addition of TPGS (Fig. 3I, J, and L).

10.1128/mSphere.00564-18.1 FIG S1 The effect of norfloxacin and 0.2 mM liposoluble vitamin E combination against *Burkholderia* strains, using MHB at 24 h (*n* = 6, 3 independent repeats). Results are shown as mean MIC ± SEM, using paired *t* test. Download FIG S1, TIF file, 0.4 MB. Copyright © 2018 Naguib and Valvano. 2018 Naguib and Valvano This content is distributed under the terms of the Creative Commons Attribution 4.0 International license.

10.1128/mSphere.00564-18.2 FIG S2 The growth curve of wild-type (A) and Δ*bcnA* (B) strains which are challenged with sub-MIC of norfloxacin, with or without TPGS. *n* = 6. Download FIG S2, TIF file, 0.4 MB. Copyright © 2018 Naguib and Valvano. 2018 Naguib and Valvano This content is distributed under the terms of the Creative Commons Attribution 4.0 International license.

Together, the combined results of the experiments in this section demonstrate that vitamin E, in either soluble or liposoluble forms, exerts an adjuvant effect enhancing the bactericidal activity of different antibiotics in a BcnA-dependent manner.

### Vitamin E prevents BcnA antibiotic binding.

**FIG 4:**
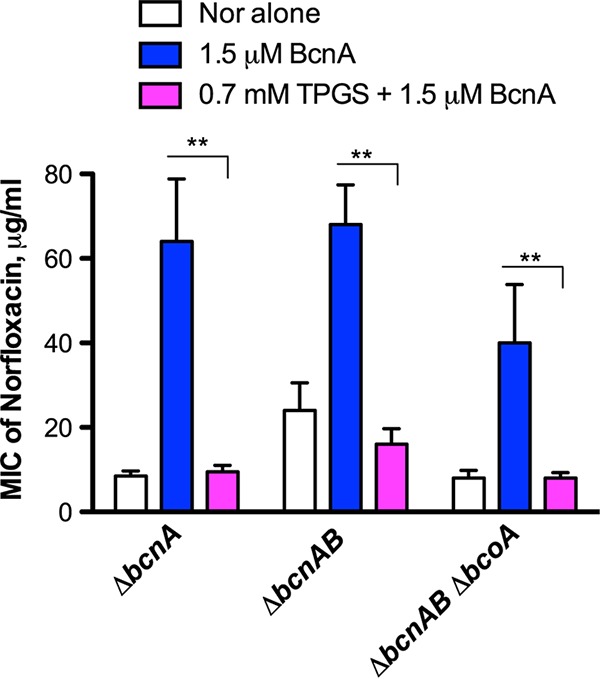
*In vitro* protection assays against norfloxacin with 1.5 μM BcnA protein in the absence or presence of 0.7 mM TPGS. The results were obtained from 4 independent experiments, each in duplicate (*n* = 8), and plotted as the mean MIC ± SEM. **, *P = *0.02 determined by paired *t* test.

To determine the mechanism of the vitamin E antibiotic adjuvant effect, we performed chemical complementation experiments in which 1.5 μM purified BcnA was added to the various Δ*bcnA* mutants in the presence or absence of the antibiotic and with or without TPGS. The chosen amount of BcnA was the minimal protein concentration that gives an effect in Galleria mellonella and mouse infections, as described previously (7). Exogenously added BcnA protein conferred increased norfloxacin resistance to Δ*bcnA,* Δ*bcnAB*, and Δ*bcnAB* Δ*bcoA* mutants (MIC values of 64, 76, and 32 μg/ml, respectively), but this effect was abolished in the presence of TPGS, resulting in MICs of 8 μg/ml for the three strains (*P = *0.02) (Fig. 4). Similar results were also obtained using the Δ*bcnA* mutant treated with norfloxacin, 0.2 mM liposoluble vitamin E, and 1.5 μM BcnA protein; the MIC decreased from 32 ± 11.31 μg/ml to 4 ± 0 μg/ml (*P = *0.04). In this experiment, the MIC of norfloxacin was reduced by 8-fold compared to the treatment with BcnA protein only (*P = *0.04). Together, these experiments support the idea that TPGS and liposoluble vitamin E interact with BcnA protein directly and prevent antibiotic capture by BcnA.

**FIG 5:**
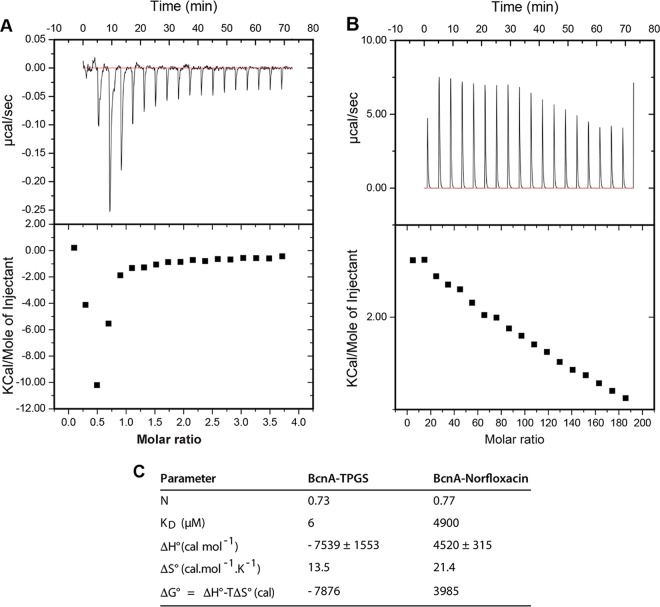
Binding analyses by microcalorimetry. (A and B) ITC data of TPGS binding to BcnA (A) and the presence of norfloxacin (B). The bottom panel shows that the binding isotherm is obtained by plotting the areas under the peaks in the top panel against the molar ratio of ligand (TPGS or norfloxacin) added to BcnA present in the cell. (C) Physicochemical binding parameters of TPGS and norfloxacin to BcnA. The binding affinity (*K*) and enthalpy change (Δ*H*°) were obtained from ITC profiles fitting to “one set of binding sites” modeled by Origin 7 software. Δ*G*° and *T*Δ*S*° were determined by the equations Δ*G*° = − RT ln*K* and *T*Δ*S*° = Δ*H*° − Δ*G*°. *N* is the binding stoichiometry. *K_D_* is the dissociation constant. The data presented are the average from two experiments.

Binding of TPGS and norfloxacin to BcnA was investigated by isothermal titration microcalorimetry (ITC), a bioanalytical technique that provides the thermodynamics parameters of the interactions between molecules by measuring the dissipated or absorbed heat upon the binding event, allowing the calculation of association and dissociation (*K_D_*) constants and the enthalpy (Δ*H*°) and entropy (Δ*S*°) of the interaction (17–19). The binding properties of TPGS toward BcnA indicated a *K_D_* of nearly 6 μM with a stoichiometry (N value) approaching 1 (0.73) (Fig. 5C), indicating the binding of one monomer of BcnA per TPGS molecule, which is also in agreement with the hyperbolic shape of the titration thermogram (Fig. 5A). The calculated negative Δ*H*° and positive Δ*S*° values of the TPGS-BcnA interaction suggested that strong electrostatic interactions play a key role in binding, and the Δ*G*° of −7.876 kcal indicates that the binding reaction is favorable and spontaneous. The binding properties of norfloxacin toward BcnA showed a *K_D_* of 4.9 mM, with a stoichiometry of 0.77 (Fig. 5C), denoting also the binding of one monomer of BcnA per norfloxacin molecule. In contrast to the titration thermogram for TPGS, the thermogram for norfloxacin corresponded to an endothermic binding interaction with BcnA (Fig. 5B). A positive Δ*H*° (Fig. 5C) usually indicates that the complex formation between norfloxacin and BcnA is mainly driven by hydrophobic interactions (20, 21). Indeed, hydrogen bonding has been proposed as a mechanism for the binding of norfloxacin to activated carbon nanotubes (22) and polydopamine microspheres (23). The calculated dissociation binding constant for TPGS-BcnA was approximately 800-fold higher than that of norfloxacin-BcnA (Fig. 5C), consistent with the high concentration of norfloxacin required to detect binding to BcnA. Together, these results agree with previously reported molecular dynamics studies showing that norfloxacin weakly binds BcnA at the flexible loops in the rim of the BcnA cavity (7). Therefore, the chemical complementation experiments and the direct binding assays demonstrate that BcnA binds strongly to vitamin E and this high-affinity interaction prevents the much weaker BcnA antibiotic binding.

### Vitamin E treatment enhances *in vivo* survival of *Burkholderia-*infected G. mellonella larvae.

**FIG 6:**
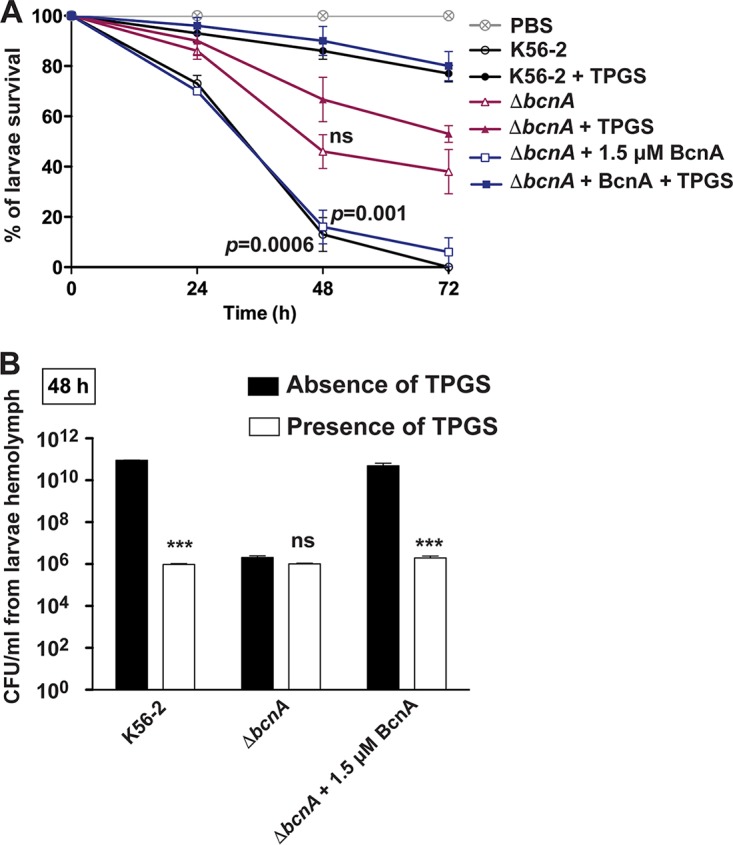
*In vivo* protection assays using G. mellonella infection model. (A) Larvae were infected with 8 × 10^3^ CFU of B. cenocepacia K56-2, Δ*bcnA* mutant, and Δ*bcnA* mutant plus 1.5 μM BcnA protein in the presence or absence of 0.7 mM TPGS. Larva survival was monitored over 72 h postinfection. (B) Hemolymph from at least three B. cenocepacia-infected larvae was extracted and pooled at 48 h postinfection to determine the bacterial CFU/ml by plating onto LB agar containing 200 μg/ml spectinomycin (to prevent the growth of the larvae’s endogenous microbial flora agar. The results represent the mean and the SEM from 3 independent biological replicates. ***, *P* < 0.001.

We reported previously that coadministration of BcnA and *Burkholderia* to G. mellonella larvae increases the bacterial load and reduces larvae survival. Because infected G. mellonella larvae mount a cellular and humoral response characterized by the production of multiple antimicrobial peptides (24, 25), these results suggested that BcnA can bind and neutralize antimicrobial peptides (in a similar way as polymyxin B). We employed the G. mellonella infection model to probe the effect of the BcnA-inhibitory activity of TPGS after infection with the parental strain and the Δ*bcnA* mutant using a dose of 8 × 10^3^ CFU for each strain. At 48 h postinfection, both the parental K56-2 strain and the Δ*bcnA* mutant chemically complemented by coinjection of 1.5 μM BcnA caused great mortality resulting in less than 20% larva survival and no larvae surviving at 72 h (Fig. 6A). As expected, the control experiment using the Δ*bcnA* strain showed survival rates of 45% and 40% at 48 and 72 h, respectively (Fig. 6A). Administering 0.7 mM TPGS together with the bacterial inoculum enhanced survival up to 90% and 80% at 48 and 72, respectively, in larvae infected with either K56-2 or the Δ*bcnA* strain plus BcnA protein (Fig. 6A). In contrast, the survival of larvae infected with the Δ*bcnA* strain was not significantly improved by coadministration of TPGS in comparison to infection with the Δ*bcnA* strain only. Analysis of the bacterial loads recovered from the larvae’s hemolymph at 48 h demonstrated that K56-2 bacterial CFU decreased up to 6 log in the presence of TPGS compared to the bacterium-only infection (Fig. 6B). Similar results were obtained with the chemically complemented mutant bacteria in the presence of TPGS, while no significant difference was observed in the bacterial counts of larvae infected with the Δ*bcnA* strain in the presence or absence of TPGS (Fig. 6B).

**FIG 7:**
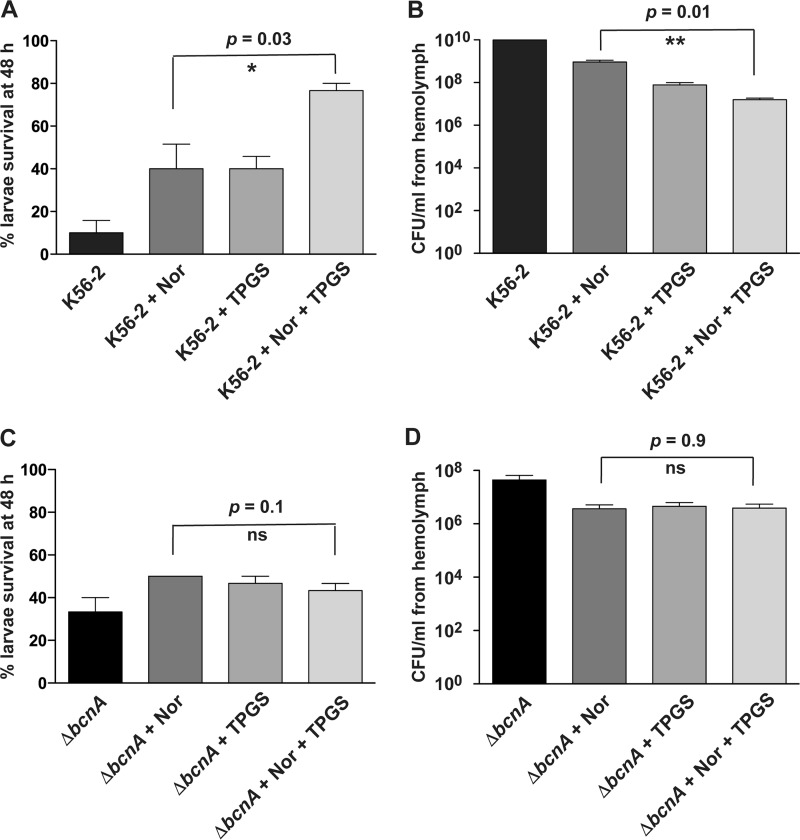
Treatment of G. mellonella*-*infected larvae with norfloxacin or norfloxacin plus TPGS in combination with the wild type or Δ*bcnA* mutant. Larvae were infected with 100 CFU of bacteria for 24 h and then treated with the MIC of norfloxacin appropriate to each bacterial strain with or without 1.7 mM TPGS. Percent survival and the bacterial load (CFU/ml) in the larva’s hemolymph were determined at 48 h postinfection. The results represent the mean and SEM for 3 biological repeats. (A and B) Percent survival and bacterial load, respectively, of larvae infected with the parental K56-2 strain. (C and D) Percent survival and bacterial load, respectively, of Δ*bcnA* strain-infected larvae.

We also investigated if the norfloxacin-TPGS combination can be applied as a treatment for previously infected G. mellonella larvae. For this experiment, larvae were infected with 100 CFU of the parental K56-2 strain or the Δ*bcnA* mutant, and treatment with norfloxacin only, TPGS only, or norfloxacin plus TPGS was given at 24 h postinfection. Compared to norfloxacin- or TPGS-only treatments, the survival of K56-2-infected larvae increased to more than 80% upon treatment with norfloxacin and TPGS (Fig. 7A), and this correlated with at least a 3-log reduction in the bacterial load (Fig. 7B). In contrast, the combination of norfloxacin and TPGS did not protect larvae against infection with the Δ*bcnA* strain (Fig. 7C and D). These results indicate that TPGS can improve the *in vivo* efficacy of an antibiotic in a BcnA-dependent manner.

### Vitamin E increases antibiotic susceptibility in P. aeruginosa.

**FIG 8:**
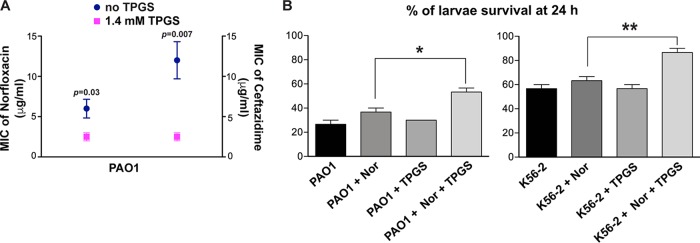
Vitamin E can reduce the antibiotic resistance of P. aeruginosa PAO1. (A) MIC of norfloxacin and ceftazidime with and without 1.4 mM TPGS against PAO1 using microdilution method at 24 h (*n* = 4, from 2 independent experiments and 2 biological replicates). Results are shown as the mean MIC ± SEM and compared using the paired *t* test. (B) Norfloxacin-TPGS treatment of G. mellonella larvae infected with 10 CFU of P. aeruginosa PAO1 or B. cenocepacia K56-2 for 2 h and then treated with the MIC of norfloxacin, 1.7 mM TPGS, or the two together. Percentage of larva survival was monitored after 24 h. *n* = 3. *, *P* < 0.05; **, *P* < 0.01.

To investigate if the adjuvant effect of TPGS can be applicable to other bacterial species, we performed *in vitro* and *in vivo* experiments using the classical P. aeruginosa PAO1 strain. *In vitro*, the combination of norfloxacin and ceftazidime with 1.4 mM TPGS showed significant reduction (over 3-fold) in the MICs of both antibiotics against the strain PAO1 in comparison to the MICs for the antibiotic-only treatments (Fig. 8A). We also performed the treatment of infected larvae using norfloxacin or the combination of norfloxacin and TPGS. Because strain PAO1 is highly virulent for G. mellonella, we used an inoculum of 10 CFU and performed the treatments at 2 h postinfection, which gives sufficient time to establish an infection (26). A statistically significant difference was seen between the survival of untreated larvae and that of larvae treated with norfloxacin and TPGS together (Fig. 8B). Similar results were obtained, as expected, in the control experiments using larvae infected with the B. cenocepacia K56-2 strain at a dose of 10 bacterial cells per larva. In this case, while the survival rate of untreated G. mellonella was 30%, more than 80% survival was obtained in larvae treated with norfloxacin and TPGS (Fig. 8B). Therefore, these results support the notion that the benefit of vitamin E as antibiotic adjuvant treatment is not specific to B. cenocepacia.

## DISCUSSION

We have recently established that production and secretion of the bacterial lipocalin BcnA, especially from bacteria exposed to near-lethal antibiotic concentrations, provide B. cenocepacia and other Gram-negative bacteria an additional mechanism of resistance against different classes of bactericidal antibiotics (5, 7). Increased resistance is based on the ability of BcnA to scavenge antibiotics in the extracellular milieu. In this study, we demonstrate that BcnA binding to water-soluble and liposoluble forms of vitamin E increases bacterial susceptibility to antibiotics both *in vitro* and during infection using the G. mellonella model. We found that the potentiating effect of vitamin E on antibiotic activity occurs only in bacteria that produce BcnA but not in mutants lacking the *bcnA* gene. We further show that vitamin E binds directly to the purified B. cenocepacia BcnA lipocalin with an affinity in low nanomolar concentrations, while the model antibiotic norfloxacin binds with an affinity in the micromolar level. These results agree with previous molecular docking predictions indicating that the alkyl chain of liposoluble vitamin E is buried into the BcnA β-barrel tunnel and that its cyclic head is placed toward the entrance of the tunnel (7). Therefore, we conclude that vitamin E-bound BcnA results in a net increase of free antibiotic concentration, explaining both the increased antibiotic susceptibility and the dependence on BcnA for this phenotype.

In addition to authentic vitamin E, we employed here TPGS, a succinylated polyethylene glycol-bound derivative of vitamin E that retains the same properties of α-tocopherol but is water soluble. Water solubility allows for more reproducible quantitative experimental conditions. Further, TPGS is already used in the pharmaceutical industry as a wetting, emulsification, solubilization, and spreading agent (16, 27). TPGS can solubilize water-soluble and lipophilic molecules, forming various types of micelles (28) and increasing the solubility of drugs like cyclosporines, taxanes, steroids, and antibiotics (reviewed in reference 16). Administration of vitamin E has been reported to enhance antibiotic activity, but the mechanism appears to be indirect. For example, administration of vitamin E prior to infection improves the *in vivo* efficacy of antibiotics in wounds experimentally infected with methicillin-resistant S. aureus (29) due to a general improvement in immunological parameters. Similarly, vitamin E administration reduces inflammation in a rat model of Escherichia coli pyelonephritis (30) and in a Streptococcus pneumoniae lung infection model (31). In contrast, our data demonstrate that the antibiotic adjuvant effect of vitamin E and TPGS, at least in B. cenocepacia and P. aeruginosa, is associated with the presence of a functional BcnA protein, thus revealing a previously unappreciated connection between a bacterial lipocalin and vitamin E binding.

Vitamin E is considered the main antioxidant in biological membranes (32), and many of its beneficial effects in immunoregulation (33) are attributed to its radical scavenging activity in lipophilic environments, resulting in the stabilization of polyunsaturated fatty acids in membrane lipids. At the same time, the physical properties of vitamin E make this molecule and its derivatives useful carriers for drug delivery in general (16) and for pulmonary drug delivery in particular (34–36). Given our results, it might be feasible to consider including vitamin E in antibiotic formulations delivered by nebulization to patients with cystic fibrosis to increase their efficacy. In summary, we conclude that vitamin E acts as a lipocalin antibiotic binding inhibitor, helping to increase the effective concentration of antibiotics around bacterial cells, suggesting that vitamin E could be used as an antibiotic adjuvant in combination with antibiotics for the treatment of infections caused by multidrug-resistant *Burkholderia* and other similar multidrug-resistant bacteria.

## MATERIALS AND METHODS

### Strains and reagents.

The bacterial strains and plasmids used in this study are listed in Table S1 in the supplemental material. Bacteria were cultured in Mueller-Hinton broth (MHB) or Luria Broth (LB) at 37°C. Antibiotics (Sigma-Aldrich, United Kingdom) were diluted in water, except for polymyxin B, which was diluted in 0.2% bovine serum albumin with 0.01% acetic acid. TPGS (Sigma-Aldrich, United Kingdom) was dissolved in water at a concentration of 140 mM. Liposoluble α-tocopherol (vitamin E; Sigma-Aldrich, United Kingdom) was dissolved in dimethyl sulfoxide (DMSO).

### Molecular biology methods.

Genomic DNA was extracted from 1-ml overnight bacterial cultures suspended in LB. After centrifugation, bacterial pellets were resuspended in 400 μl lysis buffer containing sodium dodecyl sulfate (SDS) and incubated at room temperature for 10 min. Protein and cell debris were precipitated by adding 200 μl NaCl and incubating in ice for 20 min, followed by centrifugation at 4,000 rpm for 15 min. The supernatant was precipitated with absolute ethanol and centrifuged, and the DNA pellets were resuspended in diethylpyrocarbonate (DEPC)-treated deionized water. All procedures were carried out using DEPC-treated water. Genomic DNA (gDNA) samples were stored at −20°C. RNA was extracted and purified using the RNeasy Mini Plus kit (Qiagen), as indicated by the manufacturer. RNA samples were treated with 5 μl of DNase, and 5 μl of 10× DNase buffer (Turbo DNA Free) was added to 40 μl of RNA in a 1.5-ml Eppendorf tube. Reaction mixtures were incubated at 37°C for 1.5 h, and reactions were terminated by adding 10 μl DNase inactivation reagent followed by incubation for 5 min at room temperature. DNase-treated samples were centrifuged for 5 min, and the supernatant containing the RNA was stored at −80°C. Purity and quality of the RNA samples were determined by agarose gel electrophoresis using 1% agarose gels supplemented 1:10 with Midori green. Also, RNA samples were screened for gDNA contamination after DNase treatment by PCR with 16S control primers. cDNA synthesis was carried out using the RevertAid kit according to the manufacturer’s instructions. This kit allows for initial priming of RNA samples with random primers followed by cDNA synthesis. The initial step allows for degradation of RNA secondary structure prior to reverse transcription. cDNA samples were kept at −20°C until used. PCRs were carried out with the Dream *Taq* PCR Master Mix (ThermoFisher Scientific), and the primer pairs are listed in Table S1.

### Purification of BcnA protein.

Escherichia coli BL21 bacteria harboring pET28a-*bcnA* were grown for 3 h with 500 mM isopropyl thio-β-galactoside (ThermoFisher Scientific, United Kingdom) at 25°C; bacteria were harvested, resuspended in 50 mM phosphate buffer (PBS), pH 7.4 (Sigma-Aldrich, United Kingdom), with protease inhibitors (Roche Diagnostic GmBH, Germany), and lysed using a cell disruptor at 27 kpsi (Constant Systems). The lysate was cleared by centrifugation at 16,000 × *g* for 60 min at 4°C (Sorvall RC 6 Plus, Germany), and the supernatant was filtered through 0.2-μm in-line filters. The supernatant was passed through a HisTrap FF 5-ml column, using AKTA Start (GE Healthcare Bio-science, Sweden). The purified protein was detected by Coomassie blue staining following 16% SDS-PAGE and quantified by Bradford assay using bovine serum albumin (BSA) as standard.

### Checkerboard assays.

TPGS or α-tocopherol was combined with different concentrations of norfloxacin, ceftazidime, or polymyxin B, and the effect of each combination was tested against the parental B. cenocepacia K56-2, various mutant strains and their corresponding complement strains (as indicated in Results), and also Pseudomonas aeruginosa PAO1. In the combination studies, the final ranges of drug dilutions used were 2 to 128 μg/ml for norfloxacin and 2 to 512 μg/ml for ceftazidime, as recommended by the CLSI for MICs of antibiotics tested by broth microdilution (37). The final ranges of TPGS were 0.175 to 2.8 mM, and those for liposoluble α-tocopherol were 8 to 1,024 μg/ml. Inocula were prepared by diluting an overnight broth culture of each of the selected isolates in double-strength cation-adjusted MHB to reach a final inoculum of 5 × 10^5^ CFU/ml. One honeycomb plate was used for each isolate. Each well received 50 μl of the antibiotic under test, 50 μl of the vitamin E form, and 100 μl of the inoculated double-strength broth. Plates were incubated at 37°C with continuous shaking in a Bioscreen C (MTX Lab Systems, Vienna, VA) for 24 h. The MIC endpoint was read as the lowest concentration of antibiotic at which the percent OD_600_ relative to no-antibiotic control was ≤10%, which corresponded to no visible growth.

### *In vitro* antibiotic challenge assays.

Challenge assays were performed as previously described (38) with few modifications. Bacterial cultures with an OD_600_ of 0.0002 in MHB were challenged with MIC_25_ of norfloxacin (for 2 h) or polymyxin B (for 2 and 6 h) in either the absence or presence of 0.7 mM TPGS and incubated at 37°C at 180 rpm. Samples were withdrawn and serially diluted in PBS. Then, 10-μl aliquots were spotted onto the surface of MH agar plates. The plates were incubated at 37°C for 24 h, and the resulting colonies were counted.

### *In vitro* antibiotic protection assays.

Overnight cultures of the mutants (Δ*bcnA*, Δ*bcnAB*, and Δ*bcnAB* Δ*bcoA*) in MHB were diluted to an optical density at 600 nm of 0.005 in fresh cation-adjusted MHB containing different concentrations of norfloxacin and 1.5 μM BcnA protein, with or without 0.7 mM TPGS, and incubated at 37°C with medium by continuous shaking in a Bioscreen C. Bacterial growth was assessed turbidimetrically at 600 nm. The MIC endpoint was read as the lowest concentration of norfloxacin at which the percent OD_600_ relative to no-antibiotic control was ≤10%, which corresponded to no visible growth.

### Isothermal titration calorimetry.

ITC experiments were performed on a MicroCal iTC200 microcalorimeter (Malvern, United Kingdom). The sample cell was rinsed twice with 330 μl PBS (pH 7.4) before one rinse with approximately 330 μl of purified BcnA protein solution, which was left soaking for at least 2 min. The sample cell was reloaded with the protein solution (0.03 mM), and the syringe was loaded with ligand solution (TPGS or norfloxacin), purged, and refilled two times. Ligand concentration in the cell ranged from 20- to 1,000-fold greater than that of protein. Run parameters were as follows: 18 injections, initial delay of 60 s, spacing of 240 s, filter period of 5 s, injection volume of 2 μl, cell temperature of 25°C, reference power of 10 μcal/s, stirring speed of 1,500 rpm, and low feedback mode. The blanks included PBS buffer with the ligand solution. Data were analyzed with the Origin software provided by the instrument manufacturer.

### Galleria mellonella larva infection.

G. mellonella larvae were acquired from UK Waxworms Ltd., stored in wood shavings in the dark at 16°C prior to infection, and used within 2 weeks of receipt. Larvae of approximate weights of 250 to 350 mg were used. Larval infection assays were performed as previously described (39, 40). Bacteria were grown in 5 ml LB, harvested during exponential phase, resuspended in sterile PBS, and serially diluted. Larvae were injected with 10 μl of the bacterial suspension in the absence and presence of TPGS, containing approximately 8 × 10^3^ CFU of either K56-2 wild type (WT) or Δ*bcnA* mutant or *ΔbcnA* mutant plus 1.5 μM BcnA protein, using a microliter Hamilton syringe. A group of 10 control larvae were injected with 10 μl of PBS in parallel. Larvae were incubated at 37°C in the dark, and their viability was checked at 24-h intervals over a period of 72 h, according to visual color change and lack of movement upon stimuli. Three independent experiments were performed.

In similar assays, at 48 h postinfection, hemolymph samples were collected from 10 larvae/group (approximately 100 μl) in microcentrifuge tubes containing 50 μl of a saturated solution of 1 mg/ml *N*-phenylthiourea (Sigma-Aldrich, United Kingdom) and 100 μl of 1% sodium deoxycholate (Sigma-Aldrich, United Kingdom). The hemolymph was immediately serially diluted in PBS and then inoculated on LB agar plates supplemented with spectinomycin at 100 μg/ml and incubated at 37°C for 24 h, after which colonies were counted. Three independent experiments were routinely performed.

For antibiotic treatment of infected G. mellonella larvae, overnight cultures were diluted in PBS, pH 7.4, to a final concentration of 10^2^ CFU of either the K56-2 or Δ*bcnA* strain. The larvae were injected with 10 μl of the bacterial suspensions or sterile PBS (10 larvae/group in each experiment) with a Hamilton syringe and incubated at 37°C in the dark for 24 h. Then, larvae were treated with the MIC of norfloxacin, 1.4 mM TPGS, or the two together and again kept at 37°C in the dark, and their viability was checked. After 48 h postinfection, hemolymph was pooled and CFU were determined by viable count technique using 200 μg/ml spectinomycin agar. The same method was used for Galleria mellonella larvae infected with 10 CFU of PAO1 or K56-2 for 2 h only and then treated with the MIC of norfloxacin, 1.7 mM TPGS, or the two together.

### Statistical analyses.

All statistical analyses were conducted with GraphPad Prism 5.0. The results are indicated as the mean ± the standard error of the mean (SEM). The paired *t* test was used to compare the means of two unmatched groups. A paired *t* test was used to compare the means of two matched groups. Experiments were conducted with a minimum of three biological repeats, each with at least 2 technical repeats.

10.1128/mSphere.00564-18.4 TABLE S2 Bacterial strains and plasmids. Download Table S2, PDF file, 0.03 MB. Copyright © 2018 Naguib and Valvano. 2018 Naguib and Valvano This content is distributed under the terms of the Creative Commons Attribution 4.0 International license.
