# Supplementary material for: Vitamin E Increases Antimicrobial Sensitivity by Inhibiting Bacterial Lipocalin Antibiotic Binding
Source: mSphere. 2018 Dec 12;3(6):e00564-18. doi: 10.1128/mSphere.00564-18 (PMC6291622; doi:10.1128/mSphere.00564-18)
Supplement: TABLE S1 [file sph006182724st1.pdf]

Table S1. Primers used in this study

Target Gene or Intergenic region	Sequence	Sense	Product Length
BACL 3310	GGCACGATCAAGTTCGACGG	Forward	189
	GTAGTCGAGGCCGAAATCGT	Reverse	
BCAL 3311	ACAAGTACAACGTGACCGGC	Forward	167
	GATCGACGTGTCCTTCCACT	Reverse	
BCAL 3312	CGAACATCCCGGTCGTCTAT	Forward	200
	AAAGGGAAGCATGCGCGAC	Reverse	
BCAL 3310-11	CTCGCGGCGCTCAAG	Forward	142
	GACGCGACAAGCGCC	Reverse	
BCAL 3311-12	CGGACGAAGTGCAGATCAAG T	Forward	171
	AAAGCCGAGAACGACAGCGA	Reverse	
	TATAATCAGCCGCCACGGCA	Reverse	
16S Control	GTAAAGCGTGCGTAGGTGGT	Forward	195
	CTTTCGTGCCTCAGTGTCAG	Reverse	
