# Supplementary figures and images for: Vitamin E Increases Antimicrobial Sensitivity by Inhibiting Bacterial Lipocalin Antibiotic Binding
Source: mSphere. 2018 Dec 12;3(6):e00564-18. doi: 10.1128/mSphere.00564-18 (PMC6291622; doi:10.1128/mSphere.00564-18)

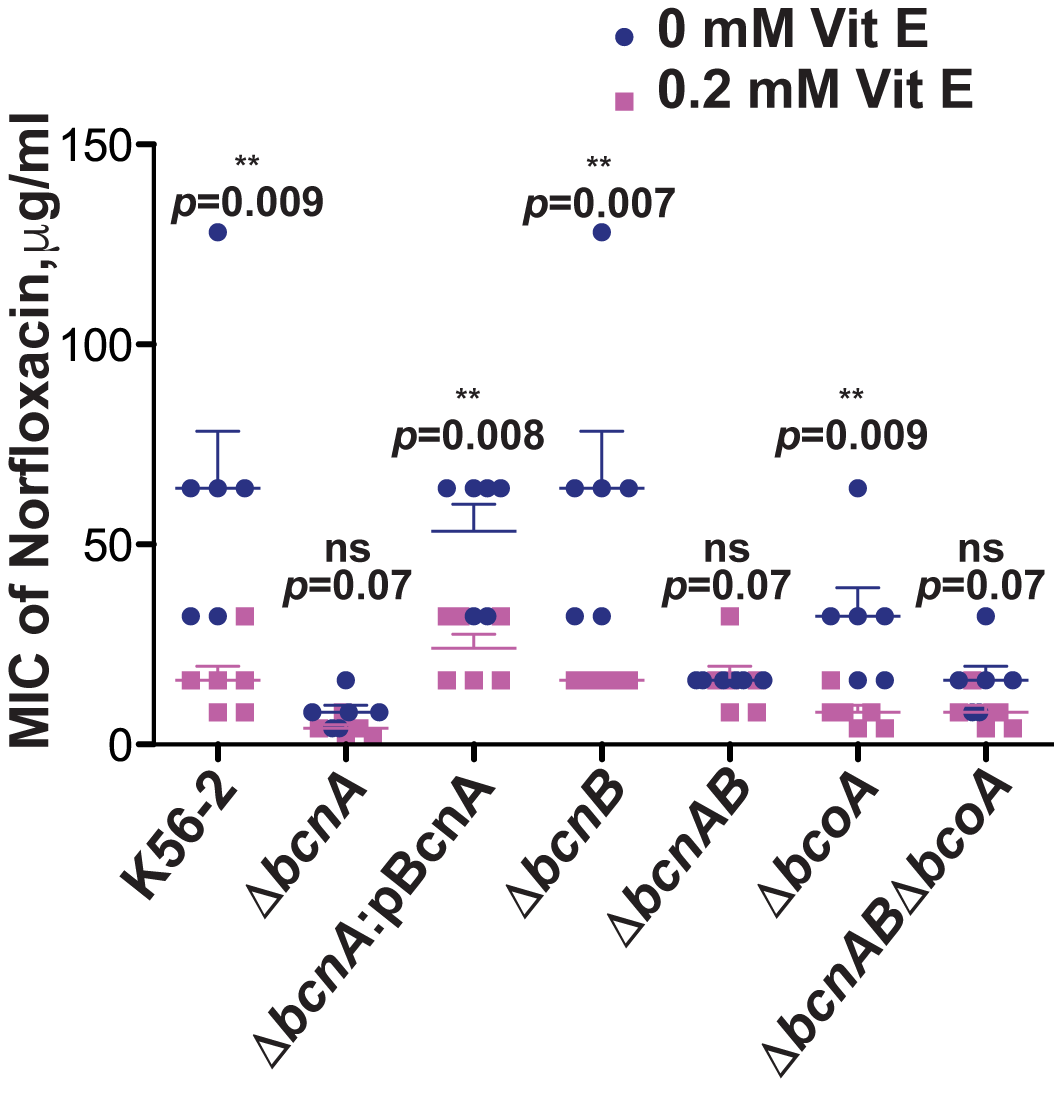

Supplement: FIG S1 [file sph006182724sf1.tif]

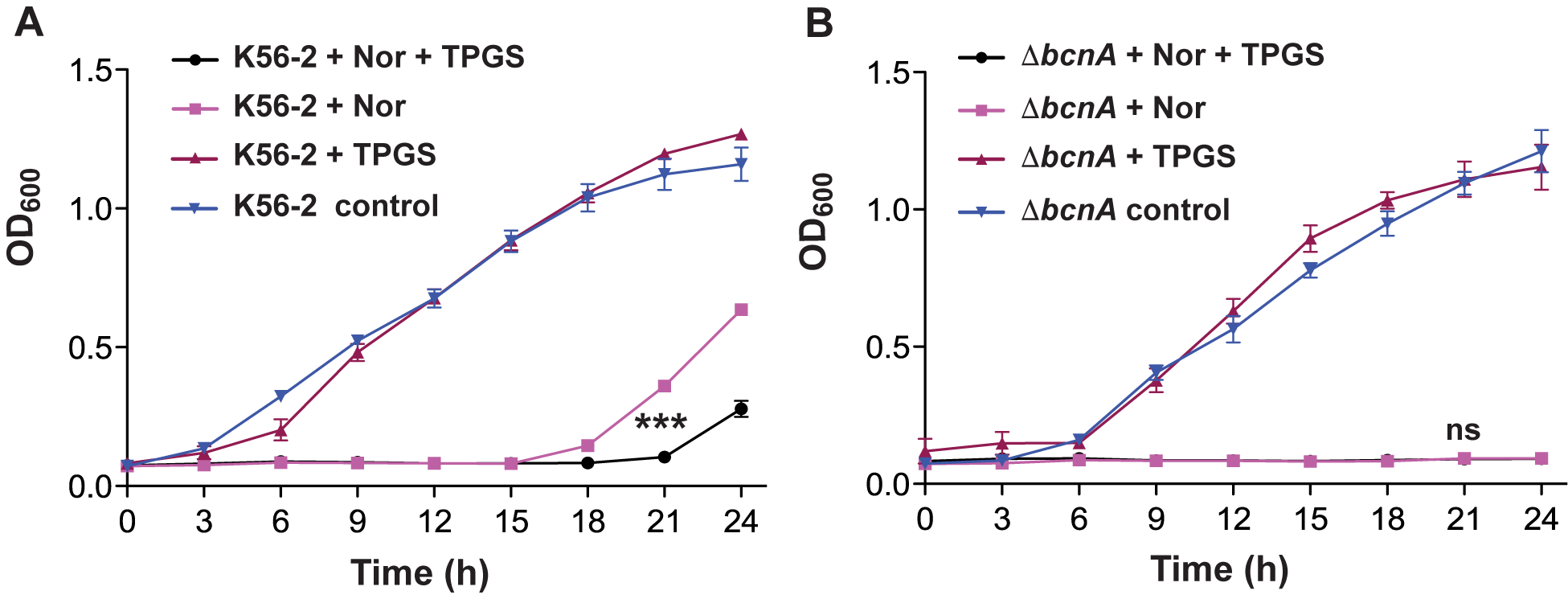

Supplement: FIG S2 [file sph006182724sf2.tif]
