# Supplementary material for: Vitamin E Increases Antimicrobial Sensitivity by Inhibiting Bacterial Lipocalin Antibiotic Binding
Source: mSphere. 2018 Dec 12;3(6):e00564-18. doi: 10.1128/mSphere.00564-18 (PMC6291622; doi:10.1128/mSphere.00564-18)
Supplement: TABLE S2 [file sph006182724st2.pdf]

Table S2. Bacterial strains and plasmids

Strains or plasmid	Relevant Characteristics[a]	Sources and/or reference[b]
**<u>Strains</u>**		
Burkholderia cenocepacia		
K56-2	ET12 clone related to J2315, CF clinical isolate	BCRRC[c] (1)
ΔbcnA	OME62, Deletion of *bcnA* gene in K56-2	(2)
ΔbcnB	OME65, Deletion of *bcnB* gene in K56-2	(2)
ΔbcnAB	OME4, Deletion of *bcnA* and *bcnB* in K56-2	(3)
ΔbcoA	OME63, Deletion of *bcoA* gene in K56-2	(2)
ΔbcnAB ΔbcoA	OME64, Deletion of *bcnA*, *bcnB* and *bcoA* in K56-2	(2)
Δ*bcnA* pDA17	MMN1, K56-2 Δ*bcnA* carrying pDA17; Tet^R	This study
Δ*bcnAB* pDA17	MMN2, K56-2 Δ*bcnB* carrying pDA17; Tet^R	This study
Δ*bcnAB*Δ*bcoA* pDA17	MMN3, K56-2 Δ*bcnAB* Δ*bcoA* carrying pDA17; Tet^R	This study
Δ*bcnA* p*bcnA*	OME86, K56-2 Δ*bcnA* carrying *bcnA* cloned in pDA17; Tet^R	(2)
Δ*bcnAB* p*bcnA*	MMN4, K56-2 Δ*bcnA* carrying *bcnA* cloned in pDA17; Tet^R	This study
Δ*bcnAB*Δ*bcoA* p*bcnA*	MMN5, K56-2 Δ*bcnA* carrying *bcnA* cloned in pDA17; Tet^R	This study
Pseudomonas aeruginosa		
PAO1	Non-CF clinical isolate	(4)
**<u>Plasmids</u>**		
pDA17	ori_{pBBRI}, Tet^R, mob+, P_{dhfr}, FLAG epitope	(5)
pExp*bcnA*	POE16, *bcnA* gene without signal peptide encoding sequence cloned in pET28a(+)	(3)
p*bcnA*	pOE13, pDA17 harboring *bcnA* gene C-terminus FLAG, Tet^R	(2)

[a]TetR , Tetracycline resistance.
[b] References are listed in the main manuscript.
[c] BCRRC, *B. cepacia* Research and Referral Repository for Canadian CF Clinics.

References

1. Mahenthiralingam E, Coenye T, Chung JW, Speert DP, Govan JR, Taylor P, Vandamme P. 2000. Diagnostically and experimentally useful panel of strains from the *Burkholderia cepacia* complex. J Clin Microbiol 38:910-913.
2. El-Halfawy OM, Klett J, Ingram RJ, Loutet SA, Murphy ME, Martin-Santamaria S, Valvano MA. 2017. Antibiotic Capture by Bacterial Lipocalins Uncovers an Extracellular Mechanism of Intrinsic Antibiotic Resistance. MBio 8.
3. El-Halfawy OM, Valvano MA. 2013. Chemical communication of antibiotic resistance by a highly resistant subpopulation of bacterial cells. PLoS One 8:e68874.
4. Holloway BW. 1955. Genetic recombination in *Pseudomonas aeruginosa*. J Gen Microbiol 13:572-581.

5. Andrade A, Valvano MA. 2014. A *Burkholderia cenocepacia* gene encoding a non-functional tyrosine phosphatase is required for the delayed maturation of the bacteria-containing vacuoles in macrophages. Microbiology 160:1332-1345.
